# GPAT3 regulates the synthesis of lipid intermediate LPA and exacerbates Kupffer cell inflammation mediated by the ERK signaling pathway

**DOI:** 10.1038/s41419-023-05741-z

**Published:** 2023-03-24

**Authors:** Guoqiang Fan, Yanfei Li, Yibo Zong, Xiaoyi Suo, Yimin Jia, Mingming Gao, Xiaojing Yang

**Affiliations:** 1 MOE Joint International Research Laboratory of Animal Health and Food Safety, Nanjing Agricultural University, Nanjing, 210095 P. R. China; 2 Laboratory of Lipid Metabolism, Hebei Medical University, Shijiazhuang, Hebei 050017 China; 3 Key Laboratory of Animal Physiology & Biochemistry, Nanjing Agricultural University, Nanjing, 210095 P. R. China

**Keywords:** Inflammation, Fat metabolism

## Abstract

In the process of inflammatory activation, macrophages exhibit lipid metabolism disorders and accumulate lipid droplets. Kupffer cells (KCs) are the resident hepatic macrophage with critical defense functions in the pathogenesis of several types of liver disease. How dysregulated lipid metabolism contributes to perturbed KCs functions remains elusive. Here we report that glycerol-3-phosphate acyltransferase 3 (GPAT3) plays a key role in KCs inflammation response. Our findings indicate that lipopolysaccharide (LPS)-mediated inflammatory activation markedly increased lipid droplets (LDs) accumulation in KCs. This increase could be attributed to significantly up-regulated GPAT3. The loss of GPAT3 function obviously reduced KCs inflammation reaction both in vivo and in vitro, and was accompanied by improved mitochondrial function and decreased production of lysophosphatidic acid (LPA), in turn inhibiting extracellular regulated protein kinases (ERK) signaling pathway. Overall, this study highlights the role of GPAT3 in inflammatory activation of KCs and could thus be a potential therapeutic target for the treatment of inflammation-related liver disease.

## Introduction

Inflammatory liver injury results in the development of many liver diseases, such as hepatitis, hepatic fibrosis, alcohol-related or non-alcoholic disorders, and hepatoma. Kupffer cells (KCs) are unique resident macrophages of the liver and important defense cells that eliminate bacteria and toxins and play a leading role in the occurrence of endotoxic liver injury by releasing various inflammatory mediators [1–3]. Activated KCs drive the inflammatory response to liver injury by secreting several mediators that regulate inflammation and homeostasis [4]. Previously studies have reported that targeted regulation of KCs reduces the incidence of liver disease, such as steatosis and liver injury [5, 6]. As hepatic disease induced by inflammatory liver injury has become a serious global health threat, the potential regulatory factors and molecular mechanisms of KC inflammation have been emerging as a subject of interest.

Recent studies have shown that the immune system and lipid metabolism are in close coordination and cooperation [7–10]. Lipids are required by all cells, ensuring the energy and essential fatty acids necessary for cells, and maintaining basic biochemical and biophysical properties. Upon activation of the inflammatory response, macrophages rapidly induce changes to lipid metabolic and energetic homeostasis [11–13]. These perturbations of lipid metabolism in macrophages change cellular functions. For instance, previous studies have shown lipid-overloaded macrophages in adipose tissue stimulate the release of pro-inflammatory cytokines [14]. Considerable evidences have emerged suggesting that some lipid metabolism-related factors may regulate macrophage inflammation response [15–17]. Therefore, understanding the directional interactions between cellular dysregulated lipid metabolism and KCs functions in inflammation situations is very important.

To explore the relationship between KCs inflammation and lipid metabolism and clarify how changes in lipid metabolism are integrated with the signaling pathways that specify macrophage functions, we analyzed the response of KCs to LPS stimulation using transcriptome and lipidomics techniques. We identified the glycerol-3-phosphate acyltransferase 3 (GPAT3) gene related to lipid metabolism, which is highly expressed in activated KCs. We discovered a hitherto unrecognized function of GPAT3 as a regulator of Kupffer cells function that promotes the inflammatory response and mitochondrial dysfunction, and this action is dependent on LPA-mediated ERK signaling pathway. These findings demonstrate the novel function of GPAT3 manipulating KCs inflammation response. These results will contribute to therapy for inflammation-related liver disease.

## Results

### High GPAT3 expression and lipid reprogramming in inflammatory Kupffer cells

**Fig. 1:**
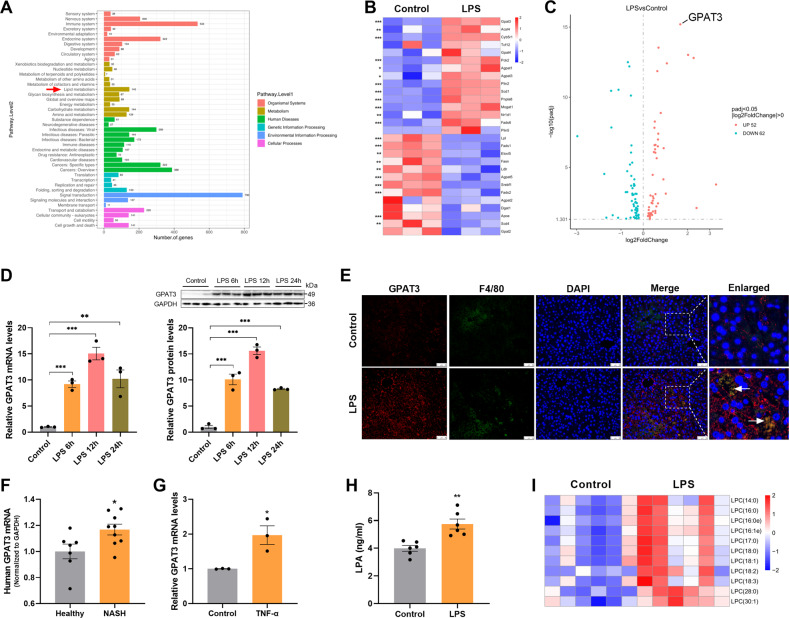
Inflammatory activation results in the high GPAT3 expression. **A** GO analysis of differentially expressed genes (DEGs) in the normal and LPS-stimulated KCs (stimulated with 1 μg/ml LPS for 24 h). DEGs were classified under six categories as indicated. Red arrow indicates lipid metabolism as the top-ranking affected metabolism pathway (*n* = 3). **B** Heatmap showing the expression of lipid metabolism pathway genes, as measured using transcriptomics, in the normal and LPS-stimulated KCs (*n* = 3). **C** Volcanic map showing the GPAT3 was the most significant difference among lipid metabolism genes after LPS stimulated KCs for 24 h (*n* = 3). **D** qPCR and Western blot analysis of GPAT3 expression in normal and LPS (100 ng/ml)-stimulated KCs at different time points (*n* = 3). **E** Immunofluorescence staining for GPAT3 expression in KCs from the LPS-treated liver mice tissues. F4/80 was used as a KCs marker. DAPI was used to visualize nuclei. Scale bars represent 50 μm (*n* = 3). **F** The mRNA expression of GPAT3 in human liver from the healthy and NASH (7 healthy human and 9 NASH patients, Database were obtained from GEO database: http://www.ncbi.nlm.nih.gov/geo/, Data set number is GSE63067). **G** GPAT3 mRNA expression in KCs after stimulated with TNF-α (100 ng/ml) for 24 h (*n* = 3). **H** LPA concentrations in the supernatant of KCs after stimulated with LPS for 24 h (*n* = 6). **I** Lipidomic analysis showing the levels of different LPC species levels in KCs (*n* = 6). Data represents mean ± SEM. ^*^P < 0.05, ^**^P < 0.01, ^***^P < 0.001.

To understand the functional significance of lipid in inflammatory KCs, we determined the transcriptomic and lipidomics profiles of KCs under LPS treatment. A Gene Ontology (GO) enrichment analysis of differentially expressed genes (DEGs) was performed to explore multiple aspects of LPS-stimulated KCs, including organismal systems, metabolism, human diseases, genetic information processing, environmental information processing, and cellular processes (Fig. 1A). Notably, metabolism is one of the main categories in the GO items and lipid metabolism was the top-ranking affected metabolic pathway, highlighting the importance of lipids among the metabolic changes triggered by LPS stimulation (Fig. 1A). We found that the expression of genes responsible for lipid metabolism changed markedly in inflammatory KCs, including genes involved in triacylglycerol (TG) synthesis and utilization (Fig. 1B). We also determined that GPAT3 was the most significantly upregulated, as shown in the volcano plot (Fig. 1C). Furthermore, we confirmed by quantitative polymerase chain reaction (qPCR) and western blot that GPAT3 increased significantly after LPS stimulation (Fig. 1D). Immunofluorescence staining further revealed that LPS treatment increased GPAT3 expression in KCs of mice livers (Fig. 1E). In addition, GPAT3 mRNA expression was significantly increased in liver of patients with nonalcoholic steatohepatitis (NASH) by GEO database analysis (Database were obtained from GEO database: http://www.ncbi.nlm.nih.gov/geo/, Data set number is GSE63067) (Fig. 1F). We also found that GPAT3 mRNA expression increased significantly after the KC inflammatory response was induced by TNF-α (Figs. 1G and S1A). GPAT3 is localized in the ER membrane, there are three other subtypes GPAT1, GPAT2 and GPAT4 [18, 19]. GPATs is the rate-limiting enzyme in the de novo pathway of glycerolipid synthesis. Our results found that the mRNA expression of GPAT1, GPAT2 and GPAT4 did not change significantly after LPS treatment in KCs, only GPAT1 decreased after LPS treatment with 12 h (Fig. S1B). These results suggested that GPAT3 may play an important role in the inflammatory process of KCs.

The lipidomics analysis revealed broad, remarkable changes in lipid composition as a result of activation of the inflammatory response, and the principal component analysis further revealed significant differences in lipids between the LPS and control groups (Fig. S1C). GPAT3 catalyzes the conversion of glycerol-3-phosphate to LPA [19]. The enzyme-linked immunosorbent assay (ELISA) results show that the secretion of LPA significantly increased after treating the KCs with LPS (Fig. 1H). Moreover, the content of lysophosphatidylcholine (LPC; LPA can be generated by catalyzing LPC) also increased significantly according to the lipidomics analysis (Fig. 1I). Plasma and hepatic TG levels increased significantly in LPS-treated mice (Fig. S1D). KCs, as the first barrier against pathogens in the liver microenvironment, also accumulated TG and LDs under LPS stimulation (Fig. S1E-H), consistent with the previous report that inflammatory macrophages increase the accumulation of lipid droplets [17, 20–22]. Taken together, our data revealed that inflammatory KCs induced high GPAT3 expression and lipids reprogramming.

### Blocking GPAT3 inhibits LPS-induced Kupffer cells inflammation

**Fig. 2:**
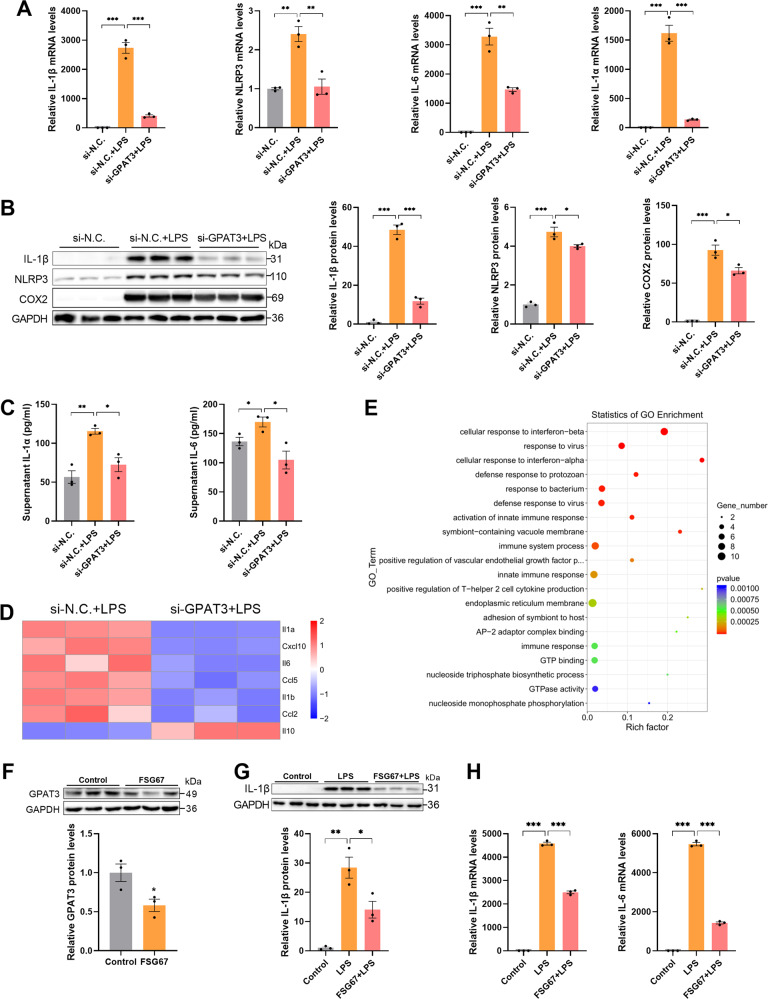
Blocking GPAT3 decreases inflammatory response in LPS-stimulated Kupffer cells. **A** The mRNA expression of IL-1α, IL-6, IL-1β and NLRP3 in si-N.C. or si-GPAT3 KCs with or without LPS (100 ng/ml, 12 h) (*n* = 3). **B, C** The protein levels of IL-1β, NLRP3, COX2, IL-1α and IL-6 in si-N.C. or si-GPAT3 KCs with or without LPS (100 ng/ml, 12 h) (*n* = 3). **D** Heatmap showing the inflammatory cytokines genes expression in LPS-stimulated KCs with or without si-GPAT3 as measured by transcriptomics (*n* = 3). **E** Scatter plot of GO enrichment differential genes of si-N.C. vs si-GPAT3 (*n* = 3). **F** The expression of GPAT3 in KCs treated with FSG67 (150 μM, 1 h) was analyzed by Western blot and qPCR (*n* = 3). **G, H** The levels of IL-1β and IL-6 in KCs were pretreated with 150 μM FSG67 for 6 h and then were treated with 100 ng/mL LPS for 6 h (*n* = 3). Data represents mean ± SEM. ^*^P < 0.05, ^**^P < 0.01, ^***^P < 0.001.

We transfected GPAT3 siRNA (si-GPAT3) into KCs to determine the role of GPAT3 in inflammatory KCs. As expected, the expression of GPAT3 mRNA and protein was significantly inhibited after transfection with si-GPAT3 (Fig. S2A, B). We next queried the effects of the loss of GPAT3 function on the LPS-induced inflammatory response. As a result, transfected GPAT3 siRNA in LPS-stimulated KCs was accompanied by a significant reduction in inflammatory capacity, as measured by interleukin (IL)-1β, NOD-, LRR- and pyrin domain-containing protein 3 (NLRP3), IL-6, IL-1α; cyclooxygenase (COX)2; and TNF-α expression (Fig. 2A–C and Fig. S2C, D). In addition, transcriptome analysis was performed on inflammatory KCs transfected with si-GPAT3; 42 genes were significantly upregulated, and 74 genes were significantly downregulated in the si-GPAT3 group compared to the si-N.C. group (Fig. S2E). Notably, we observed reductions in the expression of genes involved in the inflammatory response, including IL-1α, IL-6, and IL-1β and reductions in mRNAs encoding the chemokine (C-X-C motif) ligand 10 (Cxcl10), chemokine (C-C motif) ligand 5 (Ccl5) and Ccl2 in inflammatory KCs as a result of inhibiting GPAT3 (Fig. 2D). Gene expression of the anti-inflammatory factor IL-10 increased significantly after inhibiting GPAT3 (Fig. 2D). The Gene Ontology (GO) enrichment analysis further demonstrated that the loss of GPAT3 function significantly affected the immune response of LPS-activated KCs (Fig. 2E). FSG67 (2-(nonylsulfonamido) benzoic acid, 10-4577-Focus Biomolecules, Plymouth Meeting, PA, USA) is a GPAT inhibitor with a broad-spectrum inhibitory effect on GPAT activity [23]. The FSG67 treatment significantly inhibited the protein expression of GPAT3 (Fig. 2F). Therefore, we also used FSG67 for further study. The expression of IL-1β and IL-6 decreased significantly under the FSG67 treatment in KCs (Fig. 2G, H). Overall, these results suggest that blocking GPAT3 promoted the strong anti-inflammatory ability of activated KCs.

### GPAT3 deletion improves LPS-induced Kupffer cells mitochondrial dysfunction

**Fig. 3:**
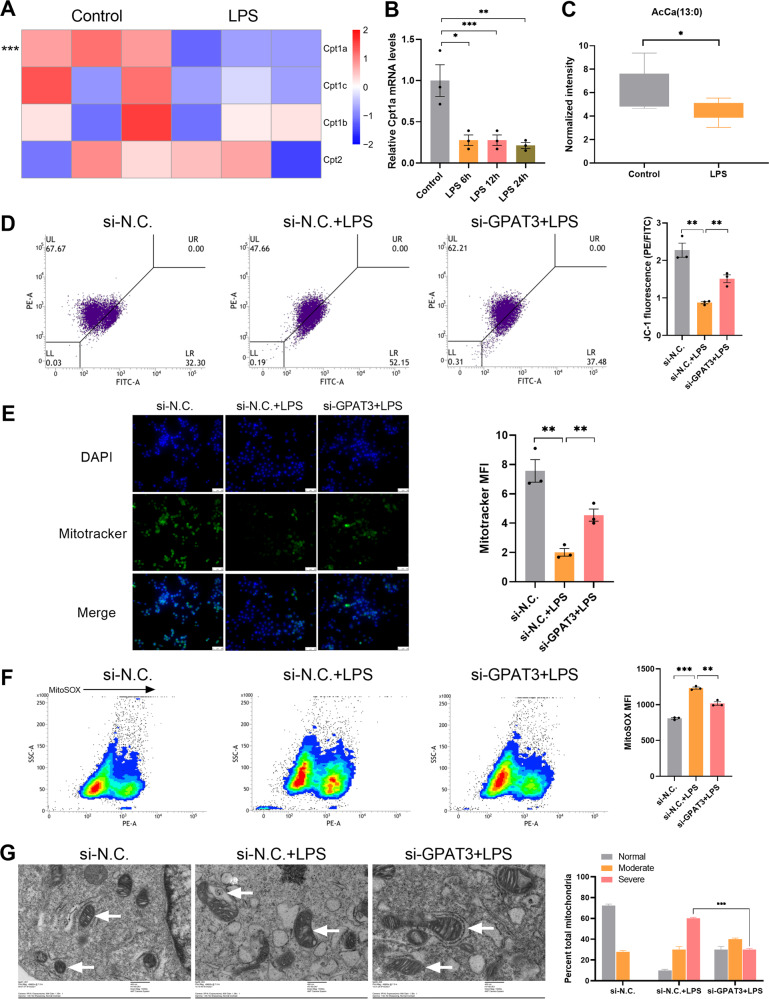
The loss of GPAT3 function improve mitochondrial function in inflammatory Kupffer cells. **A** Heatmap showing the Cpt1a, Cpt1c, Cpt1b and Cpt2 genes expression in the normal and LPS-stimulated KCs (stimulated with 1 μg/ml LPS for 24 h) (*n* = 3). **B** qPCR analysis of Cpt1a expression in normal and LPS (100 ng/ml)-stimulated KCs at different time points (*n* = 3). **C** Lipidomics data showing the AcCa (13:0) content with or without LPS (1 μg/ml 24 h) (*n* = 6). **D** The ratio of PE to FITC indicates the level of mitochondrial membrane potential. JC-1 fluorescence and **F** mitochondrial reactive oxygen species (MitoSOX) levels in si-N.C. or si-GPAT3 KCs with or without LPS (1 μg/ml, 18 h) (MFI, mean fluorescence intensity) (*n* = 3). **E** Mitotracker fluorescence visualized by fluorescence microscopy (scale bars represent 50 μm) (*n* = 3). **G** Electron microscopy of KCs and quantification of mitochondrial ultrastructural abnormalities at 18 h post stimulation with LPS without or with si-GPAT3. White arrows, mitochondria (scale bars represent 400 nm) (*n* = 3). Data represents mean ± SEM. ^*^P < 0.05, ^**^P < 0.01, ^***^P < 0.001.

Mitochondrial β oxidation involves the conversion of long-chain FA to acylcarnitine by the carnitine palmitoyltransferase 1a (Cpt1a) [24]. Cpt1a decreased in inflammatory KCs according to the transcriptome and qPCR analyses (Fig. 3A, B). Cpt1a converts long-chain FAs into acylcarnitine in the outer mitochondrial membrane, which are transported to the mitochondrial intima and finally into the matrix for FA oxidation [25]. Decreased Cpt1a was associated with a significant drop in acylcarnitine in the LPS group (Fig. 3C). We reasoned that a reduced KC inflammatory response associated with the loss of GPAT3 may be associated with changes in mitochondrial function. Therefore, we assessed mitochondrial function. The mitochondrial membrane potential (JC-1) (Fig. 3D) and mitochondrial mass (Fig. 3E) increased, respectively, in cells that lacked GPAT3 function under the LPS treatment. Mitochondrial reactive oxygen species (ROS) content (MitoSOX) was decreased in the loss of GPAT3 function after LPS treatment (Fig. 3F). Consistent with impaired mitochondrial function in inflammatory macrophages [26–28], transmission electron microscopy showed that the mitochondrial cristae in LPS-stimulated KCs were looser than resting cells, but this parameter improved by inhibiting GPAT3 (Fig. 3G). In addition, Cpt1b increased significantly in inflammatory KCs after transfection with GPAT3 siRNA (Fig. S2F). Although Cpt1b is mainly expressed in the myocardium and skeletal muscle, it is still detected in KCs. GPAT3 is located primarily in the endoplasmic reticulum (ER) [29] and is thought to be responsible for TG synthesis in different cells [30, 31]. We speculate that the ER may be affected by the loss of GPAT3 function. However, a lack of transcriptional responses within the unfolded-protein response genes Herpud1, Edem2, Bax, Eif2ak3, Bcap31, Atf6, Bak1, Serinc3, and Xbp1 indicated that GPAT3 deficiency may do not cause ER stress in LPS-activated KCs (Fig. S2G).

### GPAT3 knockout ameliorates LPS-induced hepatic injury and primary KCs inflammation in mice

**Fig. 4:**
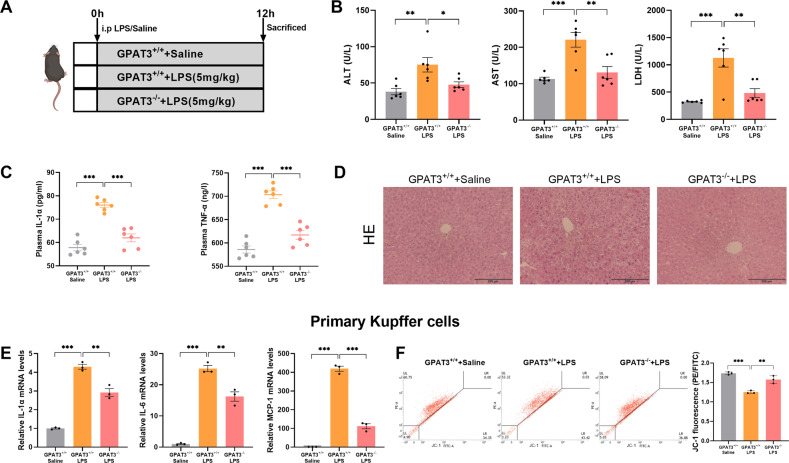
GPAT3 deletion prevents LPS-induced hepatic injury and primary KCs inflammatory response. **A** Experimental design of LPS-induced inflammation model using GPAT3^+/+^ and GPAT3^-/-^ mice. **B** Plasma ALT, AST and LDH concentration of mice (*n* = 6). **C** Plasma IL-1β and IL-6 production of mice (*n* = 6). **D** Representative images of HE staining (Scale bars represent 200 μm) in liver of mice (*n* = 3). **E** The mRNA levels of IL-1α, IL-6 and MCP-1 in primary KCs (*n* = 3). **F** Mitochondrial membrane potential in primary KCs (*n* = 3). Data represents mean ± SEM. ^*^P < 0.05, ^**^P < 0.01, ^***^P < 0.001.

To further verify the function of GPAT3, GPAT3 knockout mice were used. Plasma alanine aminotransferase (ALT), aspartate aminotransferase (AST) and Lactate dehydrogenase (LDH) were significantly decreased in GPAT3^-/-^ mice compared with GPAT3^+/+^ mice under LPS stimulation (Fig. 4B). In addition, deletion of GPAT3 inhibits LPS-induced plasma IL-1α and TNFα contents (Fig. 4C). Histologically, HE staining revealed smaller lipid droplets and less ballooning degeneration in GPAT3^-/-^ mice compared with GPAT3^+/+^ mice under LPS stimulation (Fig. 4D). These results suggest that GPAT3 KO ameliorates LPS-induced liver injury in mice. Furthermore, in primary KCs, inflammation response was significantly increased by LPS treated in GPAT3^+/+^ mice, but deletion of GPAT3 significantly inhibits the rise of inflammation (Fig, 4E). The JC-1was significantly increased in primary KCs that lacked GPAT3 function under the LPS treatment (Fig. 4F).

### GPAT3 enhances the Kupffer cells inflammation depending on the synthesis of LPA

**Fig. 5:**
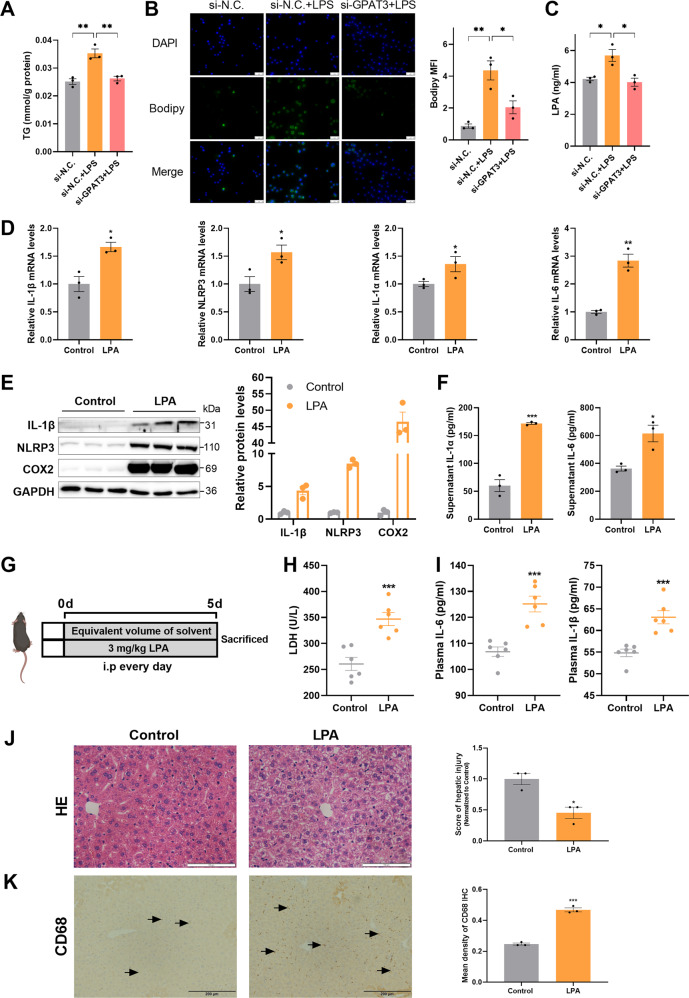
Exogenous LPA enhanced inflammatory response in vitro and vivo. **A** Effect of si-GPAT3 or si-N.C. on TG content in KCs stimulated with LPS (1 μg/ml) for 24 h (*n* = 3). **B** Bodipy 493/503 fluorescence visualized by fluorescence microscopy (scale bars represent 50 μm) (*n* = 3). **C** LPA concentrations in the supernatant of KCs after LPS (100 ng/ml 12 h) stimulated and with or without si-GPAT3 (*n* = 3). **D** The mRNA expression of IL-1β, NLRP3, IL-1α and IL-6 in KCs treated with LPA (30 μM 12 h) (*n* = 3). **E, F** The effects of exogenous LPA (30 μM 12 h) on the protein expression of inflammatory factors IL-1β, NLRP3, COX2, IL-1α and IL-6 in KCs (*n* = 3). **G** Experimental design of mice treated with LPA. **H, I** The contents of LDH, IL-6 and IL-1βin plasma of LPA treated with mice (*n* = 6). **J, K** Representative images and quantification of HE staining (Scale bars represent 100 μm) and CD68 IHC (Scale bars represent 200 μm) after LPA treatment in liver of mice (*n* = 3). Data represents mean ± SEM. ^*^P < 0.05, ^**^P < 0.01, ^***^P < 0.001.

GPAT3 is a key rate-limiting enzyme in the first step of triglyceride synthesis, the si-GPAT3 treatment reduced TG accumulation in LPS-activated KCs (Fig. 5A). Bodipy 493/503 staining showed that LD accumulation induced by LPS decreased in the si-GPAT3 treatment group compared to the si-N.C. group (Fig. 5B). Strikingly, the intermediate product of triglyceride synthesis, LPA, was decreased in the KC supernatant that lacked GPAT3 activity under the LPS treatment (Fig. 5C). This may imply that LPS-induced inflammation and abnormal mitochondrial function are related to GPAT3-mediated LPA. Next, we explored whether exogenous LPA promoted inflammatory cytokine production in KCs. Interestingly, exogenous LPA increased IL-1β, NLRP3, IL-1α, IL-6, and COX2 expression in KCs (Fig. 5D–F). In addition, i.p. injected LPA also had significant effects on inflammation in vivo. Although there was no change in body weight between the control and LPA-treated mice (Fig. S3A), LPA aggravated liver injury which showed a significant increase in plasma LDH level of mice (Fig. 5H). Moreover, the LPA treatment significantly increased the plasma levels of the inflammatory products, such as IL-6 and IL-1β (Fig. 5I). HE staining showed that inflammatory cells infiltrated in the LPA group compared to the control group (Fig. 5J). Additionally, LPA promoted the expression of proinflammatory cytokines, including IL-1α, IL-6, IL-1β, TNF-α, and NLRP3 in the liver of mice (Fig. S3B). Also, LPA promoted the accumulation of CD68 content according to the IHC staining analysis (Fig. 5K) and the increase in CD68 expression is of interest, as CD68 is strongly implicated in activated MI-type KCs [32]. Thus, our results indicate that inflammatory KCs increased the GPAT3 level to enhance the synthesis of LPA, which augmented the inflammatory activity signal.

### Mitochondrial function and the PKCθ/ERK/c-Jun pathway is involved in the LPA-induced inflammatory response

**Fig. 6:**
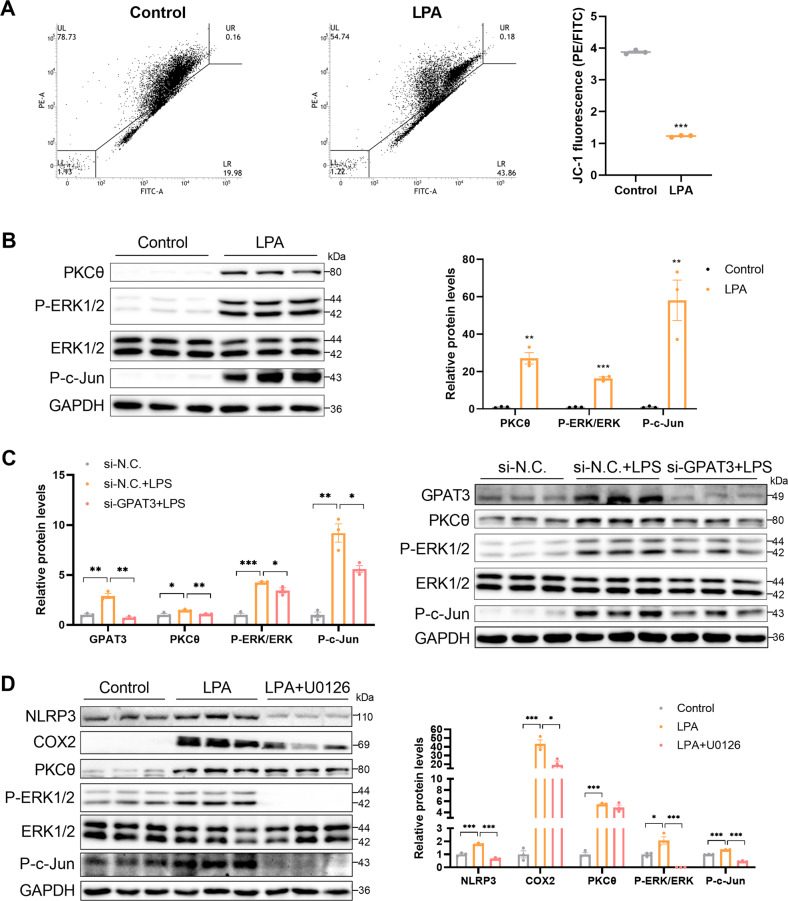
GPAT3 promotes inflammation response through the LPA-mediated PKCθ, ERK1/2 and c-Jun pathway. **A** Mitochondrial membrane potential (JC-1) in KCs treated with 30 μM LPA for 12 h (*n* = 3). **B** Western blot images and quantification of PKCθ, P-ERK1/2 and P-c-Jun protein expression in KCs treated with LPA (30 μM 12 h) (*n* = 3). **C** Western blot images and quantification of GPAT3, PKCθ, P-ERK1/2 and P-c-Jun protein expression in LPS-stimulated KCs transfected with GPAT3 siRNA (*n* = 3). **D** Western blot images and quantification of inflammatory cytokines and ERK1/2 pathway expression in control or LPA treatment KCs and pretreatment U0126 (10 μM 2 h, ERK inhibitor) (*n* = 3). Data represents mean ± SEM. ^*^P < 0.05, ^**^P < 0.01, ^***^P < 0.001.

As previously mentioned, the mitochondrial membrane potential (Fig. 3D) increased in mice lacking GPAT3 function under the LPS treatment. Here, exogenously adding LPA reduced the mitochondrial membrane potential of the KCs (Fig. 6A). This further demonstrates that the mitochondrial function affected by deleting GPAT3 was due to reduced LPA synthesis. In addition, a previous study reported that LPA promotes ROS production by activating protein kinase C (PKC) in PC-3 human prostate cancer cells [33], and LPA induces inflammation through the mitogen-activated protein kinase (MAPK) pathway [34, 35]. The expression of PKCθ, P-ERK1/2, and P-c-Jun increased significantly under the LPA treatment (Fig. 6B). In contrast, the PKCθ, P-ERK1/2, and P-c-Jun pathways were suppressed in cells that lacked GPAT3 function under the LPS treatment (Fig. 6C). Moreover, we pretreated KCs with the ERK inhibitor U0126 to investigate whether the LPA-enhanced P-ERK1/2 pathway is required for the inflammatory response. The results showed that U0126 abolished the LPA-induced KC inflammatory response, such as NLRP3 and COX2 expression (Fig. 6D). Moreover, U0126 abolished LPA-induced P-c-Jun activation, but the U0126 treatment did not affect the LPA-induced increase in PKCθ (Fig. 6D). Collectively, these findings demonstrate that GPAT3 promotes the inflammatory response through LPA-mediated activation of PKCθ, P-ERK1/2 and P-c-Jun signaling.

## Discussion

**Fig. 7:**
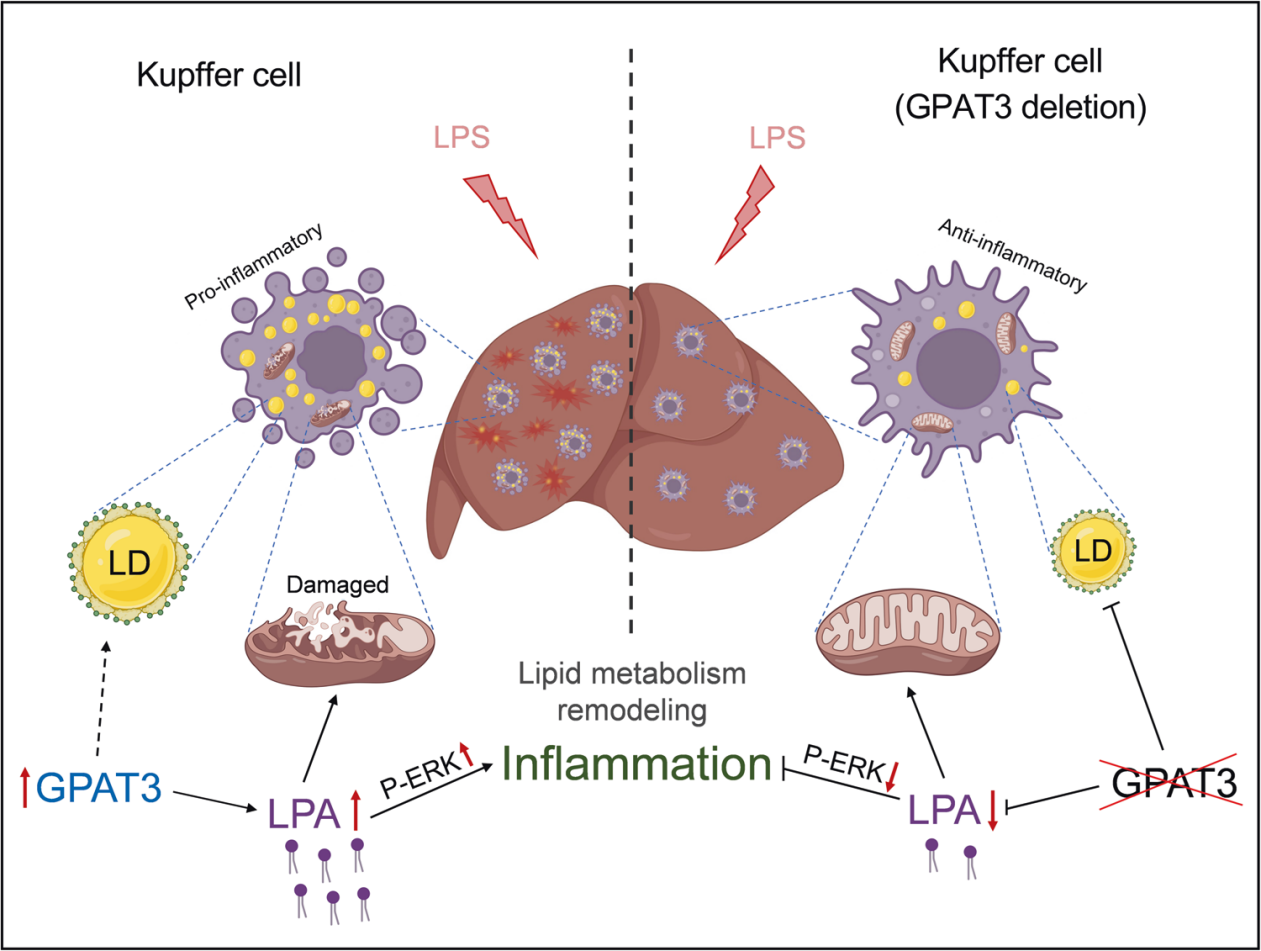
Graphical abstract. LPS induced Kupffer cells lipid metabolism remodeling and high GPAT3 expression. GPAT3 plays a critical role in the pro-inflammatory response and mitochondrial function by regulating the synthesis of LPA and up-regulate P-ERK signaling pathway.

In the present study, we identified GPAT3 as a key lipid metabolic gene involved in KCs inflammation progression. Functional experiments in vitro and in vivo demonstrated the regulatory role of GPAT3 depletion in LPS-induced KCs inflammation response. Mechanistically, GPAT3 inhibited mitochondrial function and increased the production of LPA, which promoted P-ERK signaling (Fig. 7). Therefore, our findings revealed a functional associated GPAT3 in KCs inflammation progression and identified it as a potential therapeutic target for liver injury treatment.

Accumulating reports have shown that activated macrophages change lipid composition, and that targeted regulation of fatty acid synthesis may affect the macrophage inflammatory response [17, 36, 37]. KCs are the main macrophages in the liver and are the first barrier involved in the hepatic immune response [38, 39]. Although hepatic inflammatory injury is a huge public health burden worldwide, how dysregulated lipid metabolism contributes to perturbed KC function remains unclear. Previous studies have reported that TLRs lead to the accumulation of TG in activated macrophages through a variety of pathways [40] and that different proinflammatory stimuli lead to reshaping of the lipid in macrophages in a signal-specific manner [11, 16, 17]. Our data show that the LPS treatment resulted in lipid reprogramming in KCs, meanwhile, activated KCs showed an accumulation of LDs. Transcriptome analysis showed that lipid metabolism was the most affected subset of the entire metabolism category in response to LPS stimulation. GPAT3 is localized in the ER membrane, catalyzes glycerol-3-phosphate (G3P) to produce LPA, and is the rate-limiting enzyme for the first reaction in the TG synthetic pathway [29, 41, 42]. GPAT3 plays an important role in intestinal and hepatic lipid homeostasis, dietary lipid absorption, and the production of intestinal hormones [43, 44]. Moreover, loss of function of GPAT3 alleviates insulin resistance and hepatic steatosis in seipin^-/-^mice (a mouse model of severe congenital generalized lipodystrophy) [45]. Although these studies suggest that GPAT3 plays an important role in regulating lipid homeostasis, the role of GPAT3 in the inflammation of macrophage remains unclear. In this study, we demonstrated that GPAT3 was the most significantly upregulated gene among 52 upregulated lipid metabolic genes detected in the transcriptome of KCs. Our functional study using knockdown and inhibitor FSG67 confirmed the pro-inflammatory effect of GPAT3 in vitro; that is, deleting GPAT3 significantly inhibited LPS-induced KCs inflammation. Furthermore, knockout GPAT3 also had significant effects on inflammation in vivo, and LPS-induced inflammation diminished significantly in GPAT3 KO mice. Whatever, more detailed analyses on the role of GPAT3 in liver inflammation may require studies using KC type-specific GPAT3-deficient mice.

Inflammatory-activated macrophages convert FA to TG and store it in LDs while reducing mitochondrial oxidation [22]. LPS treatment of macrophages decreases Cpt1a expression [40, 46]. Consistent with this finding, our results show that Cpt1a expression decreased significantly in LPS-stimulated KCs. Inflammatory KCs had decreased acylcarnitine contents, which was correlated with decreased Cpt1a expression. Cellular acylcarnitine level is a signature of altered mitochondrial function [47, 48], suggesting that the function of GPAT3 is related to mitochondria in inflammatory KCs. Our findings indicate that mitochondrial function improved, including the mitochondrial membrane potential, MitoSOX, and mitochondrial mass in cells that lacked GPAT3 function under the LPS treatment. In support of this notion, we observed mitochondrial structure by transmission electron microscopy (TEM) and found that the mitochondrial cristae in LPS-stimulated KCs were looser, but this parameter improved by inhibiting GPAT3. Like these results, the GPAT inhibitor FSG67 enhances palmitate oxidation of hypothalamic neurons and increases ATP contents [49].

Previous studies have shown that acute and chronic inflammation increases LPA concentration in the mouse brain [34]. Our results show that LPS-stimulated KCs increased secretion of LPA, while LPA was produced by catalyzing glycerol-3-phosphate with GPAT3, suggesting that the function of GPAT3 in inflammatory KCs may depend on the production of LPA. To test this hypothesis, we examined LPA levels in the supernatant of inflammatory KCs with loss of GPAT3 function. As expected, LPA content decreased significantly. LPA increases ROS production and induces the expression of inflammatory cytokines [33, 50]. In our study, the results showed that exogenous LPA promoted the inflammatory response in vitro and an i.p. injection of LPA also had significant effects on inflammation in vivo. An i.p. injection of LPA aggravated liver injury, which was manifested as a significant increase in the plasma LDH level, HE-stained inflammatory cell infiltration, and upregulation of the KC marker CD68. Furthermore, we explored the mechanism of GPAT3 dependence on LPA and found that exogenous LPA promoted the PKCθ, P-ERK1/2 and P-c-Jun signaling pathways. Meanwhile, this pathway was suppressed in KCs that lacked GPAT3 function under the LPS treatment. These results are consistent with previous reports that LPA induces inflammation through the PKC/MAPK pathway [33–35].

In conclusion, our current study delineates a previously undiscovered function of GPAT3 in the inflammatory response, and mitochondrial dysfunction. Inflamed KCs trigger the upregulation of GPAT3 closely followed by reprogrammed lipid metabolism properties, and the effect of GPAT3 on the inflammatory response depended on LPA production. LPA is a key link between the elevated GPAT3 levels and a KC lipid metabolic disorder that drives the development of inflammation in KCs. Increasing evidence supports the notion that different inflammatory signals reprogram lipid metabolism in macrophages, making lipids an excellent target for inflammatory therapy [51–53]. Our discovery suggests that targeting GPAT3 may be an effective therapeutic strategy for regulating the inflammatory response of KCs and improving inflammatory liver disease.

## Materials and methods

### Animals and treatments

GPAT3 knockout (KO) mice with a C57BL/6 J background were generated using CRISPR/Cas9 system, and the sgRNA targeting sites were designed on exon 2 [45]. At 8 weeks of age, GPAT3 KO and WT mice were intraperitoneally injected with 5 mg/kg LPS (L2880-Sigma, St. Louis, MO, USA). After 12 h, the plasma and liver were collected and the primary KCs were isolated from liver for subsequent analyses.

C57BL/6 J male mice (age 6–8 weeks) were purchased from Yangzhou University Comparative Medical Center. All mice were housed at 22 ± 1 °C, under a 12 h light/12 h dark cycle and fed at the Animal Experiment Center of Nanjing Agricultural University. The mice were allowed to adapt to their environment for one week. All mice had free access to water and food. In the first experiment, mice were intraperitoneally injected with 5 mg/kg LPS, plasma and liver were collected 12 h post-LPS injection to detect the content of TG and GPAT3. In the second experiment, mice were intraperitoneally injected with 3 mg/kg LPA (L7260-Sigma) or an equivalent volume of solvent (1% BSA, control) once a day for the LPA experiments, after 5 days, the plasma and liver were collected for subsequent analyses.

All animal experiments were approved by the Animal Ethics Committee of Nanjing Agricultural University, China. Euthanasia and sampling procedures complied with the “Guidelines on the Ethical Treatment of Experimental Animals” (2006) No. 398 published by the Ministry of Science and Technology, China, and with the “Regulations Regarding the Management and Treatment of Experimental Animals” (2008) No. 45, published by the Jiangsu Provincial People’s Government.

### Cell culture and transfection

Kupffer cells (KCs) were obtained from the BeNa Culture Collection (BNCC340733, Beijing, China), and were cultured in Roswell Park Memorial Institute 1640 medium (cat no. 350-000-CL, Wisent, Nanjing, China) containing 10% fetal bovine serum and 1% penicillin/streptomycin (Gibco, Grand Island, NY, USA) at 37 °C in a 5% CO_2_ atmosphere.

Specific GPAT3 small-interfering RNA (siRNA) was synthesized by GenePharma (Shanghai, China) for GPAT3 knockdown, and the sequences of the GPAT3 siRNAs were: sense (CAAGGAGUCAGCUCUUAAATT), antisense (UUUAAGAGCUGACUCCUUGTT). GPAT3 siRNA was transfected into KCs using the JetPRIME® transfection reagent (Polyplus Transfection, Beijing, China). Scrambled siRNA was used as the negative control (si-N.C.).

### Lipidomics

Liquid chromatography-mass spectrometry (LC-MS) and the data analysis were performed by BioNovoGene Co., Ltd. (Suzhou, China). Briefly, 10^7^ cells were collected, quickly frozen in liquid nitrogen, and the lipids were extracted with chloroform/methanol (2/1, v/v). LC-MS was carried out using an Acquity UPLC® BEH C18 (100 × 2.1 mm, 1.7 µm, Waters, Milford, MA, USA) column on a Thermo Ultimate 3000, and then using the Thermo Q Exactive Focus mass spectrometer. Data were analyzed with LipidSearch software.

### Transcriptomics

Total RNA of KCs was isolated and purified using TRIzol reagent (Invitrogen, Carlsbad, CA, USA). The amount and purity of the RNA in each sample were quantified using the NanoDrop ND-1000 (NanoDrop Technologies, Wilmington, DE, USA). The mRNA library was constructed and sequenced by BioNovoGene Co., Ltd. and LC-Bio Technology CO., Ltd. (Hangzhou, China). The differentially expressed mRNAs with fold changes >2 or fold changes <0.5 were selected with a *p*-value <0.05 using the R packages edgeR or DESeq2, followed by GO enrichment analyses of the differentially expressed mRNAs.

### Isolation of primary Kupffer cells

Primary KCs isolation was performed as previously described [54]. Briefly, the liver was perfused with 10 mL of phosphate-buffered saline and then digested with 0.1% type IV collagenase. Following digestion, the liver homogenate was filtered through a 75 μm stainless steel wire mesh to remove undigested tissue. The cell suspension was centrifuged at 50 *g* (Eppendorf 5810 R, Germany) for 5 min at 4 °C. The top suspension was separated with 60% Percoll and then centrifuged at 2500 *g* for 25 min. The darker layer in the middle-comprised KCs.

### Total RNA isolation and quantitative polymerase chain reaction (PCR)

Total RNA was isolated using TRIzol reagent (Tsingke, Beijing, China). A 1 μg portion of RNA was reverse transcribed to cDNA using the Reverse Transcription Master Kit (Vazyme, Nanjing, China) according to the manufacturer’s instructions. Two microliters of diluted cDNA (1:20, *v*/*v*) were used for qPCR with the Mx3000P Real-Time Polymerase Chain Reaction (PCR) System (Stratagene Inc., La Jolla, CA, USA). GAPDH was chosen as the reference gene. All primers were synthesized by Tsingke (Beijing, China). The qPCR primer sequences are listed in Table S1.

### Total protein extraction and western blot analysis

KCs were lysed in RIPA buffer (50 mM Tris-HCl pH 7.5, 150 mM NaCl, 1% NP40, 0.5% Na-deoxycholate, and 0.1% SDS) containing the complete EDTA-free and PhosSTOP protease inhibitor cocktail (Bimake, Houston, TX, USA). The protein concentration was determined following the manufacturer’s protocol for the BCA Protein Assay kit (TransGen Biotech, Beijing, China). A total of 30–50 μg of protein was used for 10% sodium dodecyl sulfate-polyacrylamide gel electrophoresis, which was transferred to a nitrocellulose membrane. The antibodies used for the western blot analysis are listed in Table S2. Images were captured using the Tannon-5200 (Shanghai, China) and band density was analyzed using Image J software. GAPDH was used as a loading control for these specific proteins.

### Flow cytometry

KCs were incubated with 3 μM BODIPY (D3922, ThermoFisher, Waltham, MA, USA), JC-1 (C2005, 1:1,000; Beyotime, Beijing, China) or MitoSOX™ Red (M36008, ThermoFisher, 1:1,000) for 30 min, respectively. Data were acquired by flow cytometry on the BD FACSVerse (BD Biosciences, Brea, CA, USA) and analyzed with the BD FACSuite.

### Fluorescence microscopy

KCs were stained with 3 μM BODIPY™ 493/503 for 20 min and then fixed in 4% paraformaldehyde for 30 min. KCs were stained with 200 nmol/L MitoTracker^TM^ Green FM (Molecular Probes, Invitrogen, Sunnyvale, CA, USA) for 30 min. Subsequently, these cells were stained with DAPI for 5 min and observed by fluorescent microscopy. Image J software was used to analyze the mean fluorescence intensity (MFI) of each image and MFI = sum of fluorescence intensity in the region /Area of the region.

Liver sections were dewaxed and antigen repaired with citrate buffer solution. Each section was soaked with in Tris-buffered saline containing 0.3% Triton X-100 for 1 h, blocked with 10% goat serum, and incubated with the primary antibody GPAT3 (20603-1-AP, Proteintech, Wuhang, China) and F4/80 (11-4801-82, Invitrogen, USA) overnight at 4 °C and then with the secondary antibody. DAPI was used as a marker for cell nuclei. Images for immunofluorescence staining were captured using a fluorescence microscope.

### Detection of TG content

TG content was measured using the Tissue/Cell Triglyceride (TG) Assay Kit (E1013, Applygen, Beijing, China) according to the manufacturer’s instructions.

### Enzyme-linked immunosorbent assay

IL-1α, IL-1β, IL-6 and TNF-α (Jiangsu Meimian Industry Co., Ltd., China) and LPA (MyBioSource, San Diego, CA, USA) levels were determined using ELISA kits according to the manufacturer’s protocols.

### Plasma biochemical measurements

Plasma TG, ALT, AST, and LDH levels were measured using an automatic biochemical analyzer (7020, Hitachi, Tokyo, Japan).

### Hematoxylin and eosin staining

Fresh livers were fixed in 4% paraformaldehyde and then paraffin-embedded. The sections were soaked and stained in Harris alum hematoxylin for about 5 min, and then washed in alcohol containing 0.5% hydrochloric acid for 10 s. After washing, the samples were soaked and dyed in eosin for 30 s, then dehydrated, transparent and sealed with neutral balsam. Finally, the morphology of the liver was examined under a microscope.

### Immunohistochemistry

Liver tissues were fixed in 4% paraformaldehyde for 24 h, paraffin-embedded, and sectioned at 5 mm. The tissue was dewaxed in xylene and antigen repair was performed by boiling the sections in citric acid buffer for 15 min, cooling for 20 min, and soaking in 3% hydrogen peroxide for 15 min. The sections were blocked in 5% goat serum before incubation with primary antibodies CD68 (BA3638, BOSTER, Wuhang, China) overnight at 4 °C. All sections were incubated with secondary antibody for 30 min before developing the color using the 3,3′-diaminobenzidine tetrahydrochloride substrate.

### Transmission electron microscopy

Fresh KCs were prepared and fixed in 0.25% glutaraldehyde, post-fixed in 1% osmium tetroxide, and embedded in resin. Ultrathin sections were cut and stained with uranyl acetate and lead citrate. The mitochondrial ultrastructure was determined with a model H-7650 transmission electron microscope (Hitachi H-7650, Hitachi Technologies, Tokyo, Japan). For quantification of mitochondrial morphological abnormality, we used the method previously reported [55]. Briefly, we assessed four parameters: electron density (light-dark); cristae swelling (tight-swollen); vacuole number (zero, one or two, three or more); and membrane damage (intact-degenerated), and we scored each of these to a scale of 1 (normal) to 3 (abnormal). Individual mitochondria were categorized as normal (score = 0–5); moderate (score = 6–9); or severe (score >10).

### Statistical analysis

All statistical analyses were performed using Prism 8 software (GraphPad Software Inc., La Jolla, CA, USA) and the results are presented as mean ± SEM. Differences were detected using either Student’s *t*-test (two-group comparison) or one-way analysis of variance (more than two groups). A *p*-value <0.05 was considered significant for all analyses. Further details on statistical analysis are listed in the figure legends.

## Supplementary information

Figure S1

Figure S2

Figure S3

Supplementary Figure legends

Supplementary tables

Reproducibility checklist

Original western blots

## Data Availability

The datasets generated and/or analyzed during the current study are available from the corresponding author upon reasonable request.
