# Supplementary figures and images for: GPAT3 regulates the synthesis of lipid intermediate LPA and exacerbates Kupffer cell inflammation mediated by the ERK signaling pathway
Source: Cell Death Dis. 2023 Mar 24;14(3):208. doi: 10.1038/s41419-023-05741-z (PMC10039030; doi:10.1038/s41419-023-05741-z)

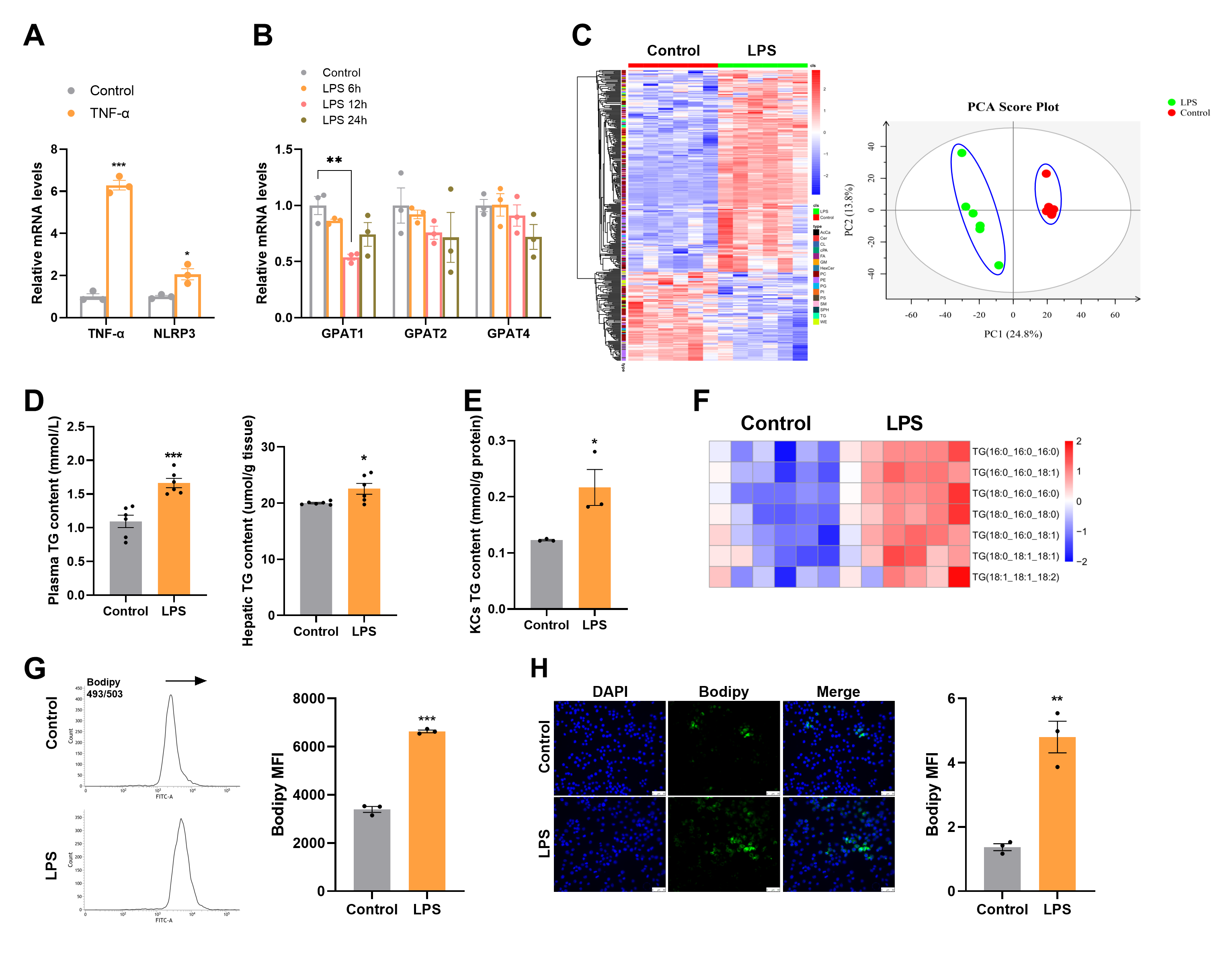

Supplement: Supplementary file 1 — Figure S1 [file 41419_2023_5741_MOESM1_ESM.tif]

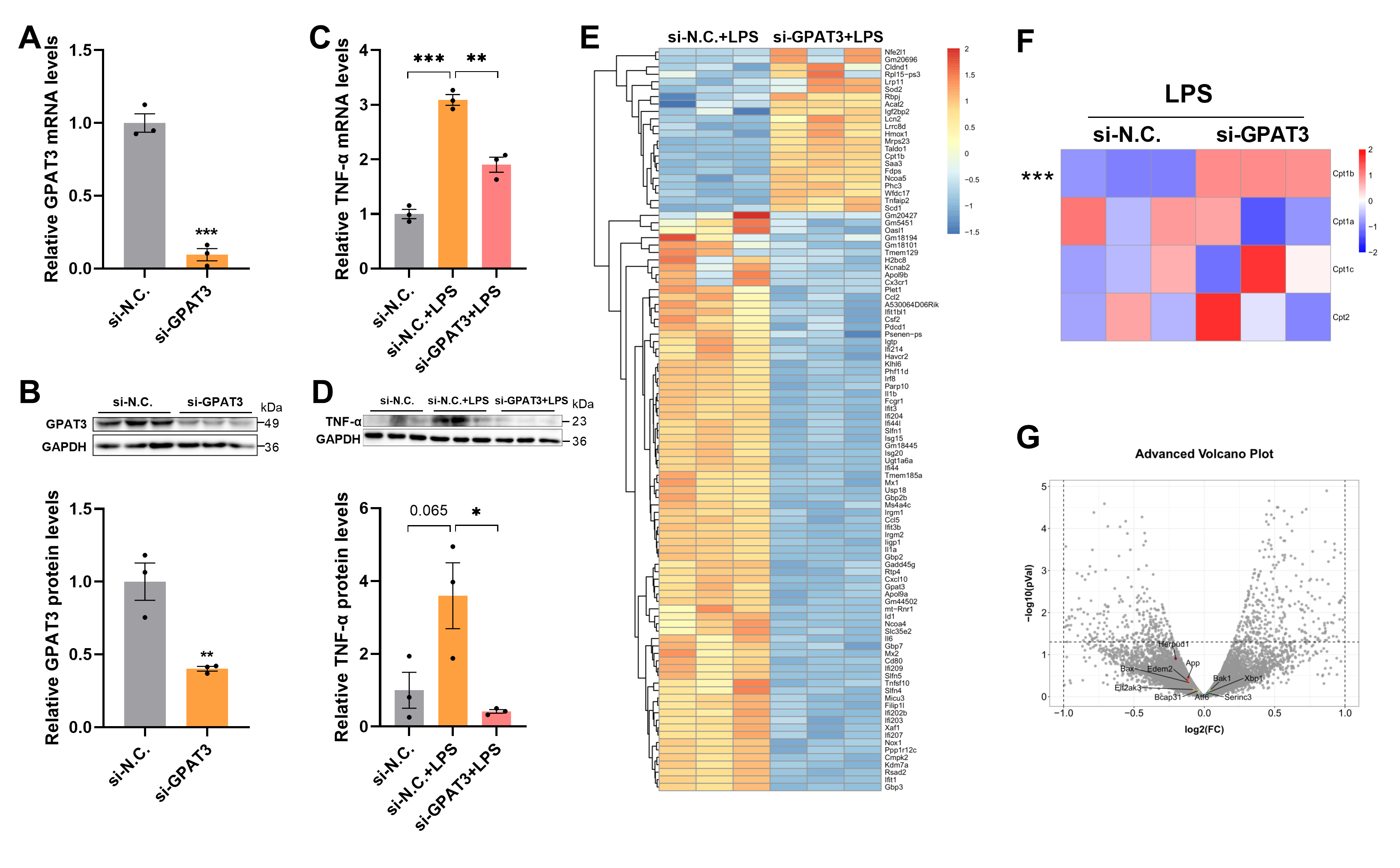

Supplement: Supplementary file 2 — Figure S2 [file 41419_2023_5741_MOESM2_ESM.tif]

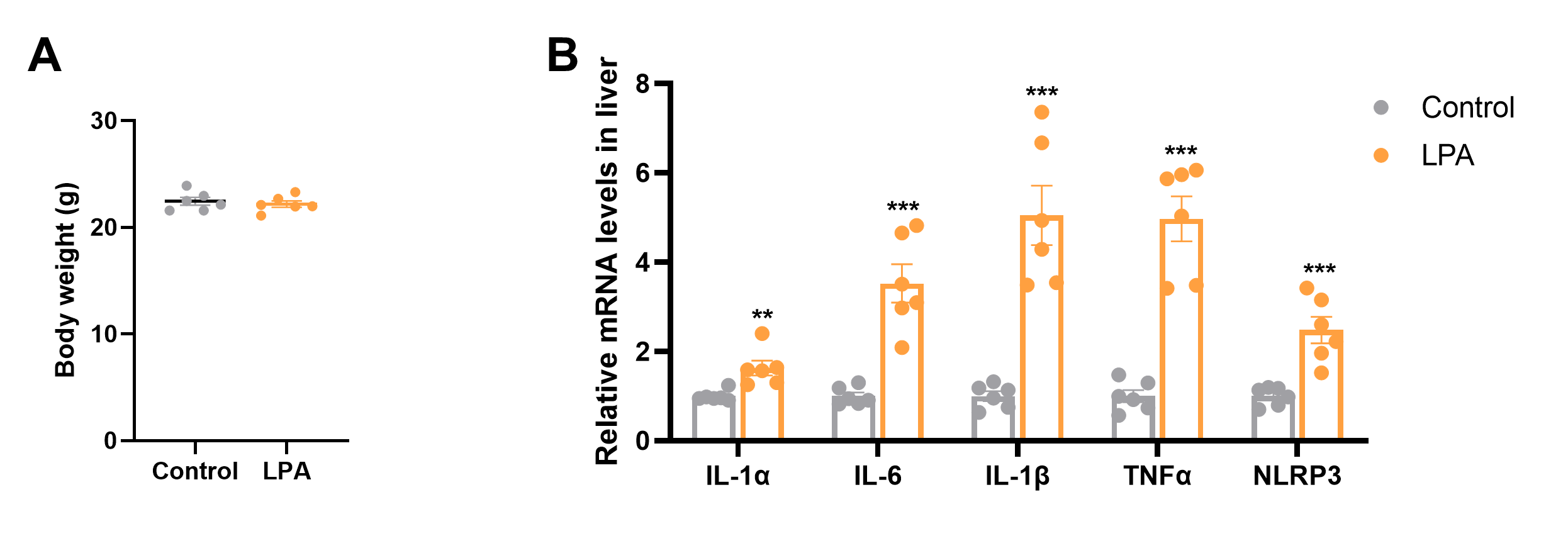

Supplement: Supplementary file 3 — Figure S3 [file 41419_2023_5741_MOESM3_ESM.tif]

Figure 1

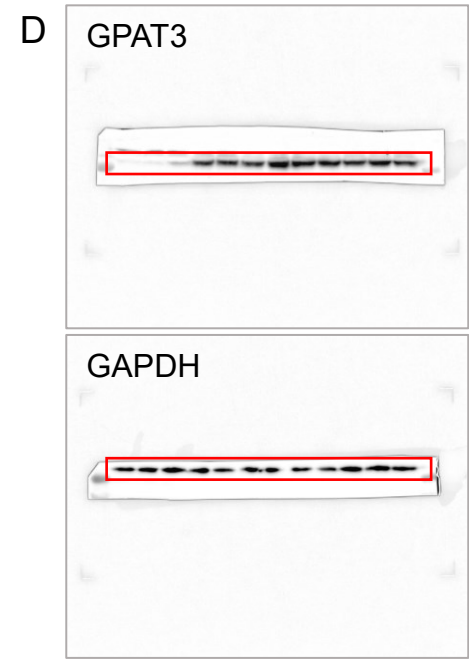

Figure 2

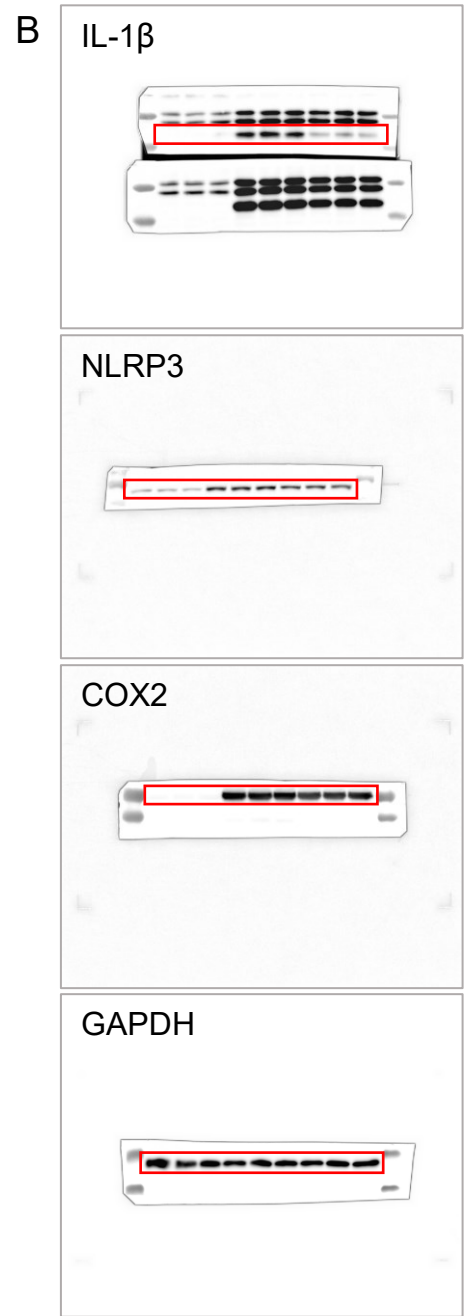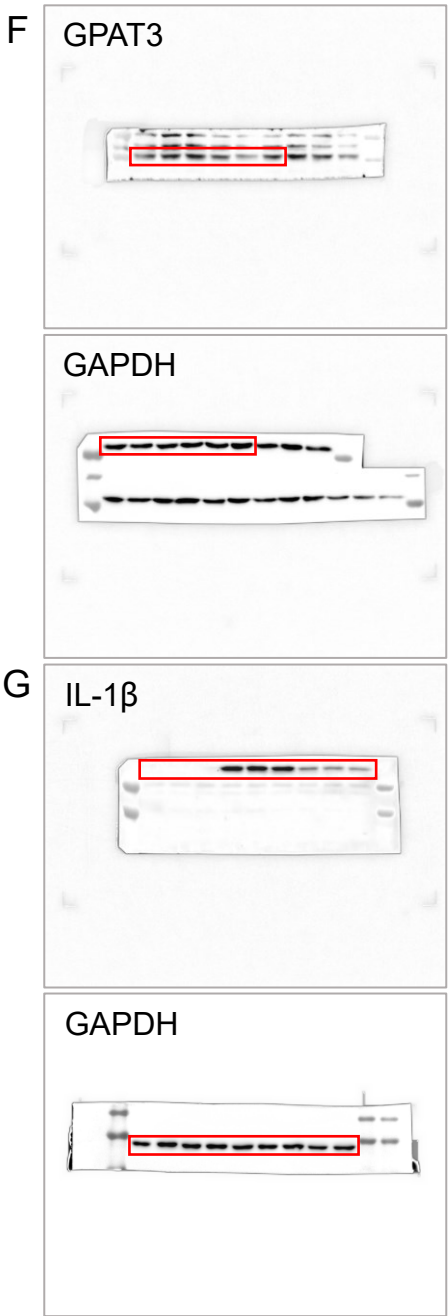

Figure 5

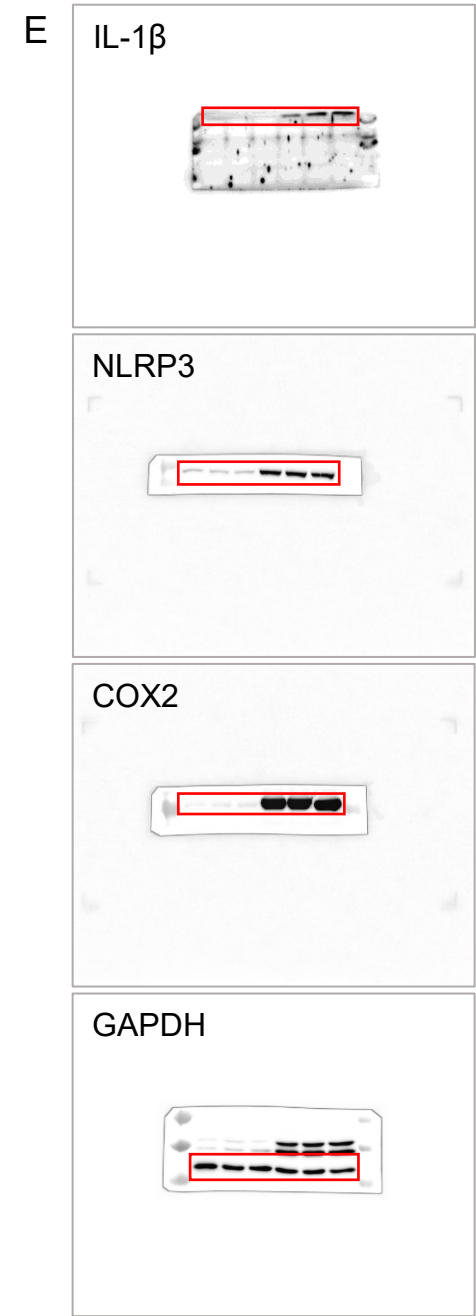

Figure 6

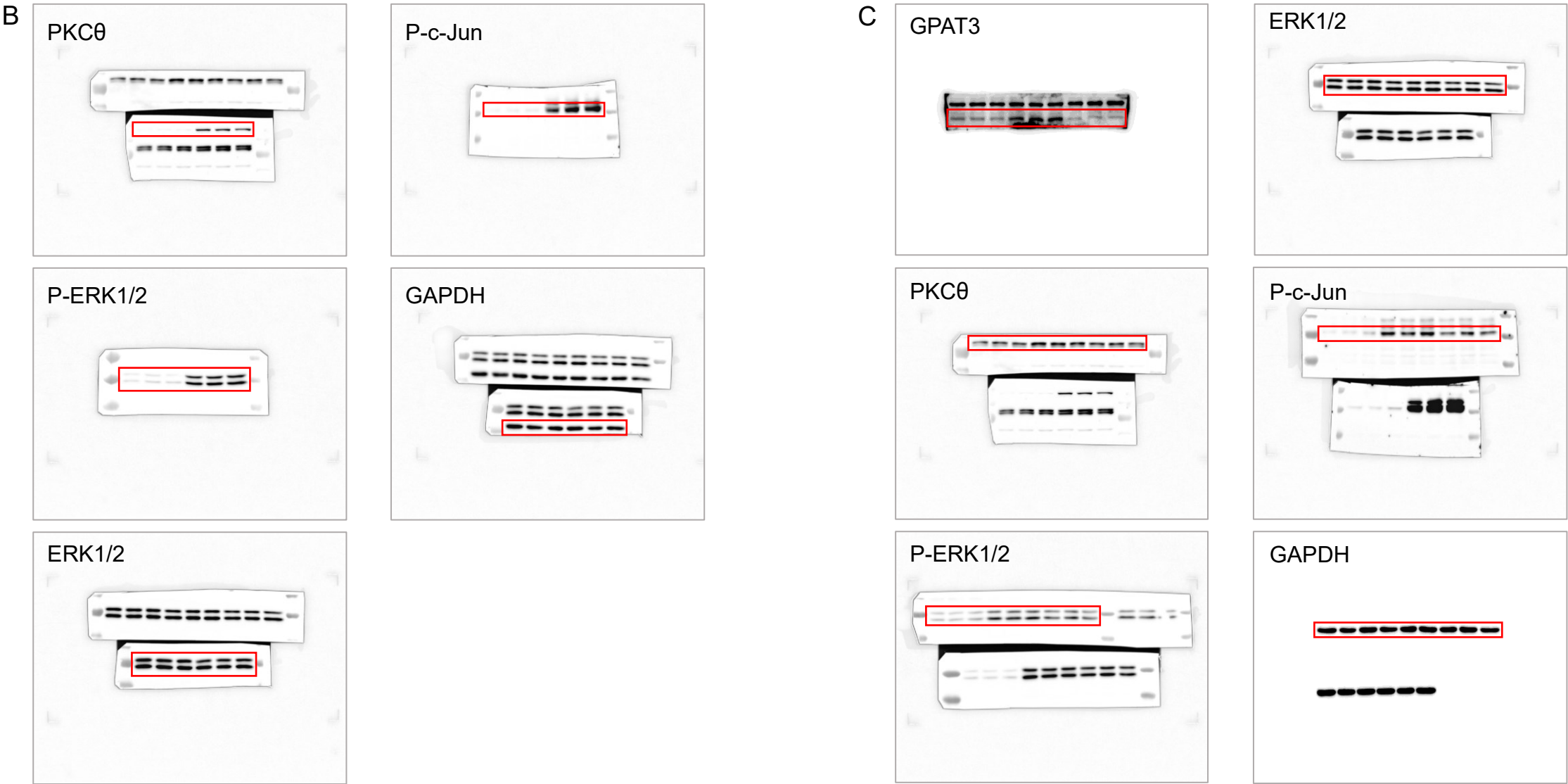

Figure 6

D

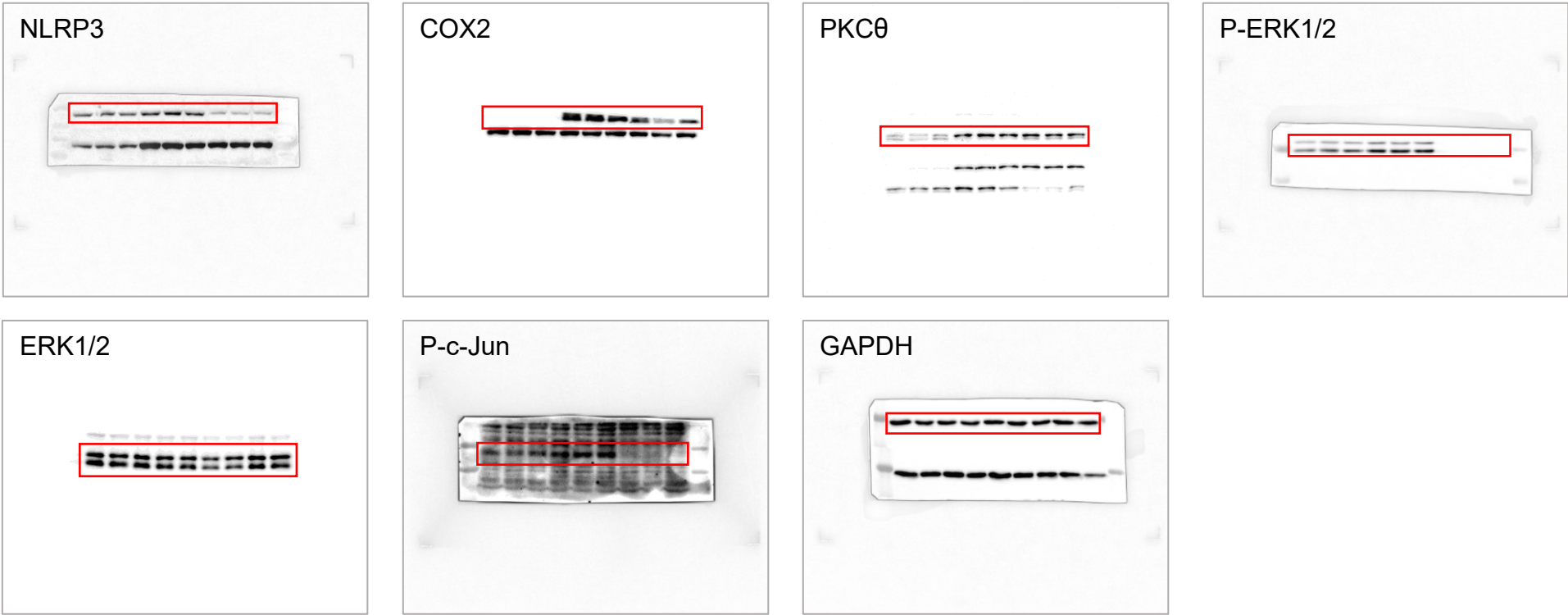

Figure S2

B

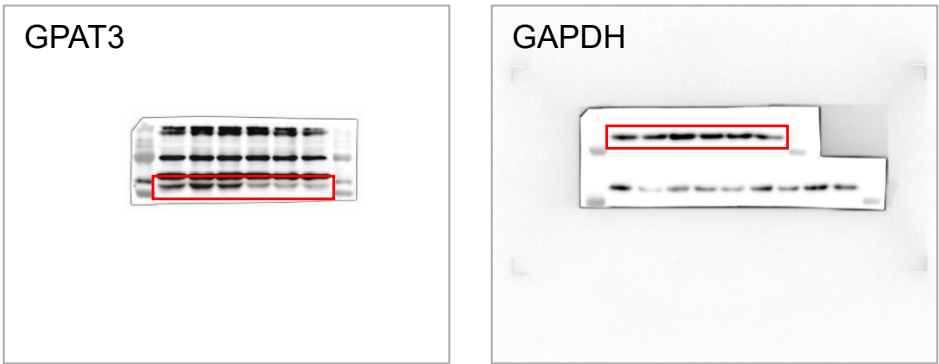

D

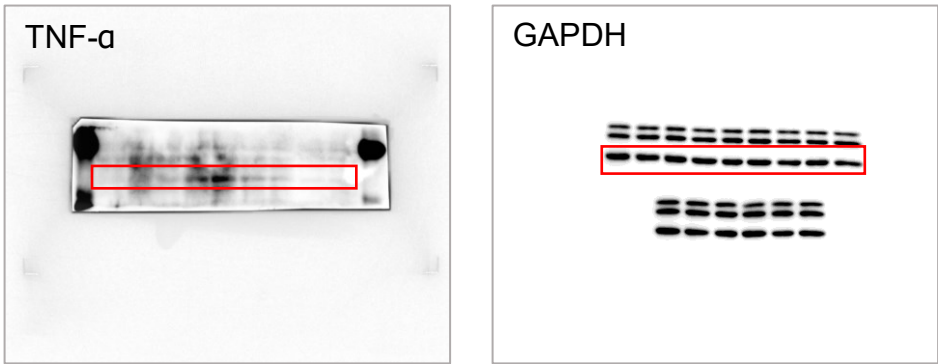

Supplement: Supplementary file 7 — Original western blots [file 41419_2023_5741_MOESM7_ESM.pdf]
