# Supplementary material for: GPAT3 regulates the synthesis of lipid intermediate LPA and exacerbates Kupffer cell inflammation mediated by the ERK signaling pathway
Source: Cell Death Dis. 2023 Mar 24;14(3):208. doi: 10.1038/s41419-023-05741-z (PMC10039030; doi:10.1038/s41419-023-05741-z)
Supplement: Supplementary file 4 — Supplementary Figure legends [file 41419_2023_5741_MOESM4_ESM.docx]

**Supplementary figure legends**

**Fig. S1** **LPS resulted in LDs accumulation and lipid reprogramming. A** The mRNA expression of TNF-α and NLRP3 in normal and TNF-α (100 ng/ml 24 h)-stimulated KCs (n = 3). **B** qPCR analysis of GPAT1, GPAT2 and GPAT4 expression in normal and LPS (100 ng/ml)-stimulated KCs at different time points (n = 3). **C** Changes in different lipid components and PCA principal component analysis of LPS vs Control in LPS (1 μg/ml, 24 h) stimulated KCs (n = 6). **D** TG levels in plasma and liver of mice injected intraperitoneally with LPS (5 mg/kg 12 h). (n = 6). **E** LPS (1 μg/ml 24 h) stimulation increased TG contents in KCs (n = 3). **F** Lipidomic analysis showing the levels of different TG species levels in KCs treated with LPS (1 μg/ml) for 24 h (n = 6). **G** Bodipy 493/503 staining in normal KCs and KCs stimulated with LPS (1 μg/ml) for 24 h, measured by flow cytometry (n = 3). **H** Bodipy 493/503 staining in normal KCs and KCs stimulated with LPS for 24 h, visualized by confocal microscopy. Scale bars represent 50 μm (n = 3). Data represents mean ± SEM. **P* < 0.05, ***P* < 0.01, ****P* < 0.001.

**Fig. S2** **Transfection efficiency of GPAT3 siRNA and changes in gene expression of inflammatory KCs as a result of loss of GPAT3 function.** **A, B** The expression of GPAT3 after transfection with GPAT3 siRNA was analyzed by qPCR and Western blot (n = 3). **C, D** The mRNA and protein expression of TNF-α in si-N.C. or si-GPAT3 KCs with or without LPS (100 ng/ml, 12 h) (n = 3). **E** Heatmap showing the genes that differ significantly in LPS-stimulated KCs with or without si-GPAT3 as measured by transcriptomics (100 ng/ml, 12 h) (n = 3). **F** Heatmap showing the Cpt1b, Cpt1a, Cpt1c and Cpt2 genes expression of KCs at 12 h post stimulation with LPS (100 ng/mL) without or with si-GPAT3. **G** ER stress response genes at 12 h post stimulation with LPS without or with si-GPAT3 as measured using transcriptomics. Data represents mean ± SEM. **P* < 0.05, ***P* < 0.01, ****P* < 0.001.

**Fig. S3** **LPA promotes the expression of inflammatory cytokines in liver of mice.** **A** Body weight in LPA-treated mice (n = 6). **B** Expression of inflammatory cytokines in LPA-treated mouse liver (n = 6). Data represents mean ± SEM. **P* < 0.05, ***P* < 0.01, ****P* < 0.001.
