# Supplementary material for: GPAT3 regulates the synthesis of lipid intermediate LPA and exacerbates Kupffer cell inflammation mediated by the ERK signaling pathway
Source: Cell Death Dis. 2023 Mar 24;14(3):208. doi: 10.1038/s41419-023-05741-z (PMC10039030; doi:10.1038/s41419-023-05741-z)
Supplement: Supplementary file 5 — Supplementary tables [file 41419_2023_5741_MOESM5_ESM.docx]

**Table S1. The primer sequences of the target genes**

| **Gene** | **Forward** | **Reverse** |
| --- | --- | --- |
| GPAT3 | TCCTTTTACCCTCGGCCTTC | AGAGCTCGAAGTCCCTTCCT |
| IL-1α | AAGAAGAGACGGCTGAGT | GTGGTGCTGAGATAGTGTT |
| IL-6 | ATGGCATCCAAGGAGTGA | GGGAGACAGAAGGGAACAG |
| IL-1β | CTTCAGGCAGGCAGTATC | CAGCAGGTTATCATCATCATC |
| NLRP3 | CCTTTGAGGCATCCAGGACAA | AAGGTTTGAGGCGGCTTTCT |
| Cpt1a | CTCCGCCTGAGCCATGAAG | CACCAGTGATGATGCCATTCT |
| TNF-α | GACGTGGAACTGGCAGAAGA | ACTGATGAGAGGGAGGCCAT |
| MCP-1 | CACAACCACCTCAAGCAC | AAGGGAATACCATAACATCA |
| GAPDH | TCTCCTGCGACTTCAACA | TGTAGCCGTATTCATTGTCA |

**Table S2. The message of antibodies for western blot**

| **Antibody** | **Company** | **Item No.** | **Dilution ratio** |
| --- | --- | --- | --- |
| GPAT3 (AGPAT9) | Proteintech | 20603-1-AP | 1:1000 |
| IL-1β | Abcam | ab254360 | 1:1000 |
| NLRP3 | Cell Signalling Technology | 15101 | 1:1000 |
| COX2 | Abcam | ab15191 | 1:1000 |
| TNF-α | Bioworld | BS5965 | 1:1000 |
| PKCθ | Bioworld | BS3248 | 1:1000 |
| P-ERK1/2 | Cell Signalling Technology | 4370 | 1:1000 |
| ERK1/2 | Cell Signalling Technology | 4695 | 1:1000 |
| P-c-Jun | Santa | sc-822 | 1:200 |
| GAPDH | Bioworld | MB001H | 1:10000 |
